# Transcriptomic analysis identified SLC40A1 as a key iron metabolism-related gene in airway macrophages in childhood allergic asthma

**DOI:** 10.3389/fcell.2023.1164544

**Published:** 2023-04-13

**Authors:** Zhili Wang, Yu He, Yupeng Cun, Qinyuan Li, Yan Zhao, Zhengxiu Luo

**Affiliations:** ^1^ National Clinical Research Center for Child Health and Disorders, Ministry of Education Key Laboratory of Child Development and Disorders, Chongqing Key Laboratory of Pediatrics, Department of Respiratory Medicine, Children’s Hospital of Chongqing Medical University, Chongqing, China; ^2^ China International Science and Technology Cooperation Base of Child Development and Critical Disorders, Chongqing Key Laboratory of Translational Medical Research in Cognitive Development and Learning and Memory Disorders, Children’s Hospital of Chongqing Medical University, Chongqing, China; ^3^ Department of Respiratory Medicine, Children’s Hospital of Chongqing Medical University, Chongqing, China

**Keywords:** asthma, childhood, data mining, iron, RNA-seq, scRNA-seq

## Abstract

**Introduction:** Asthma is the most common chronic condition in children, with allergic asthma being the most common phenotype, accounting for approximately 80% of cases. Growing evidence suggests that disruption of iron homeostasis and iron regulatory molecules may be associated with childhood allergic asthma. However, the underlying molecular mechanism remains unclear.

**Methods:** Three childhood asthma gene expression datasets were analyzed to detect aberrant expression profiles of iron metabolism-related genes in the airways of children with allergic asthma. Common iron metabolism-related differentially expressed genes (DEGs) across the three datasets were identified and were subjected to functional enrichment analysis. Possible correlations between key iron metabolism-related DEGs and type 2 airway inflammatory genes were investigated. Single-cell transcriptome analysis further identified major airway cell subpopulations driving key gene expression. Key iron metabolism-related gene SLC40A1 was validated in bronchoalveolar lavage (BAL) cells from childhood asthmatics with control individuals by quantitative reverse transcription-polymerase chain reaction (qRT-PCR) and immunofluorescence. The intracellular iron content in BAL cells was assessed by Perls iron staining and the iron levels in BAL supernatant was measured by iron assay to assess airway iron metabolism status in childhood asthmatics.

**Results:** Five common iron metabolism-related DEGs were identified, which were functionally related to iron homeostasis. Among these genes, downregulated SLC40A1 was strongly correlated with type 2 airway inflammatory markers and the gene signature of SLC40A1 could potentially be used to determine type 2-high and type 2-low subsets in childhood allergic asthmatics. Further single-cell transcriptomic analysis identified airway macrophages driving SLC40A1 expression. Immunofluorescence staining revealed colocalization of FPN (encoded by SLC40A1) and macrophage marker CD68. Down-regulation of SLC40A1 (FPN) was validated by qRT-PCR and immunofluorescence analysis. Results further indicated reduced iron levels in the BAL fluid, but increased iron accumulation in BAL cells in childhood allergic asthma patients. Furthermore, decreased expression of SLC40A1 was closely correlated with reduced iron levels in the airways of children with allergic asthma.

**Discussion:** Overall, these findings reveal the potential role of the iron metabolism-related gene SLC40A1 in the pathogenesis of childhood allergic asthma.

## Introduction

Asthma is the most common chronic airway inflammatory disease in children, with high morbidity and financial burden, accounting for 1%–2% of the healthcare budget in developed countries (Asher et al., 2006; Serebrisky and Wiznia, 2019). Studies have shown that children with early allergic sensitization, tobacco smoke exposure, as well as recurrent wheezing and severe wheezing exacerbations exhibit persistently low lung function trajectories that persist into adulthood and increase the risk of developing chronic obstructive pulmonary disease (COPD) (Berry et al., 2016; Belgrave et al., 2018). Therefore, the potential lifelong impact of poorly controlled childhood asthma on respiratory health is of great concern. Exploring the molecular mechanisms underlying childhood asthma may improve early diagnosis, prognostic evaluation, and disease control.

Childhood asthma is divided into two main phenotypes according to airway inflammation: i.e., type 2 (T2)-high allergic asthma and T2-low non-allergic asthma (Just et al., 2017; Fainardi et al., 2022). Up to 80% of asthmatic children show the allergic phenotype, which is characterized by eosinophilic airway inflammation with allergen-specific sensitization, increased blood eosinophil counts, and elevated total and specific serum immunoglobulin E (IgE) levels (Raedler et al., 2015; Fainardi et al., 2022). The primary mechanism responsible for the T2 immune response is thought to involve a complex molecular network between epithelial cell-derived alarmins [interleukin-25 (IL-25), IL-33, and thymic stromal lymphopoietin] and T2 cytokines (IL-4, IL-5, IL-9, and IL-13), mainly secreted by T2 immune cells (e.g., T helper 2 cells and T2 innate lymphoid cells) (Boonpiyathad et al., 2019; Hammad and Lambrecht, 2021). With deepening research, the roles of other cell types (e.g., macrophages and inflammatory monocytes) in allergic asthma have gradually been revealed (Draijer and Peters-Golden, 2017; Jiang et al., 2019). However, our understanding of the changes in the airway microenvironment in children with allergic asthma remains poor.

Accumulating evidence suggests that disruption of systemic and airway iron homeostasis and iron regulatory molecules may be associated with respiratory diseases, including asthma (Neves et al., 2019; Zhang et al., 2019). A longitudinal study found that lower umbilical cord iron status is associated with increased incidence of eczema and wheezing (Shaheen et al., 2004). In addition, lower exhaled breath condensate and serum iron levels are potential risk factors for childhood asthma (Vlasić et al., 2009; Ramakrishnan and Borade, 2010). Adult asthmatic patients also show reduced iron levels in bronchoalveolar lavage fluid (BALF) accompanied by increased expression of iron accumulation molecules transferrin receptor 1 (TFR1) and divalent metal transporter 1 (DMT1) in airway tissue (Ali et al., 2020). However, results in adults cannot simply be extrapolated to children due to the impact of their immature immune system and developing lungs. To the best of our knowledge, no studies on asthmatic children have explored the role of iron metabolism-related (IMR) genes.

Traditional transcriptional assays, such as bulk microarray and RNA sequencing (RNA-seq), have been widely used to identify abnormal gene expression and potential biological mechanisms in different diseases. The advent of high-throughput single-cell RNA-seq (scRNA-seq) has enabled the measurement of gene expression at single-cell resolution, opening a new avenue for understanding disease-related mechanisms (Paolillo et al., 2019; Vieira Braga et al., 2019). In the present study, we repurposed three publicly available bulk transcriptomic datasets to investigate the dysregulation of IMR genes and possible regulatory mechanisms in childhood allergic asthma. A public scRNA-seq dataset was used to identify the cell populations driving key gene expression, as well as the potential biological roles of these genes. Additionally, we performed *in silico* deconvolution analysis to identify the different cell types present in normal and allergic asthmatic airways. Finally, the expression of key IMR genes and the correlation between IMR gene expression and iron levels were validated using bronchoalveolar lavage (BAL) samples from asthmatic children and controls.

## Materials and methods

### Ethics statement

The studies involving human participants were reviewed and approved by Ethics Committee of the Children’s Hospital of Chongqing Medical University. Written informed consent to participate in this study was provided by the participants’ legal guardian/next of kin.

### Study design, participants, and settings

**FIGURE 1:**
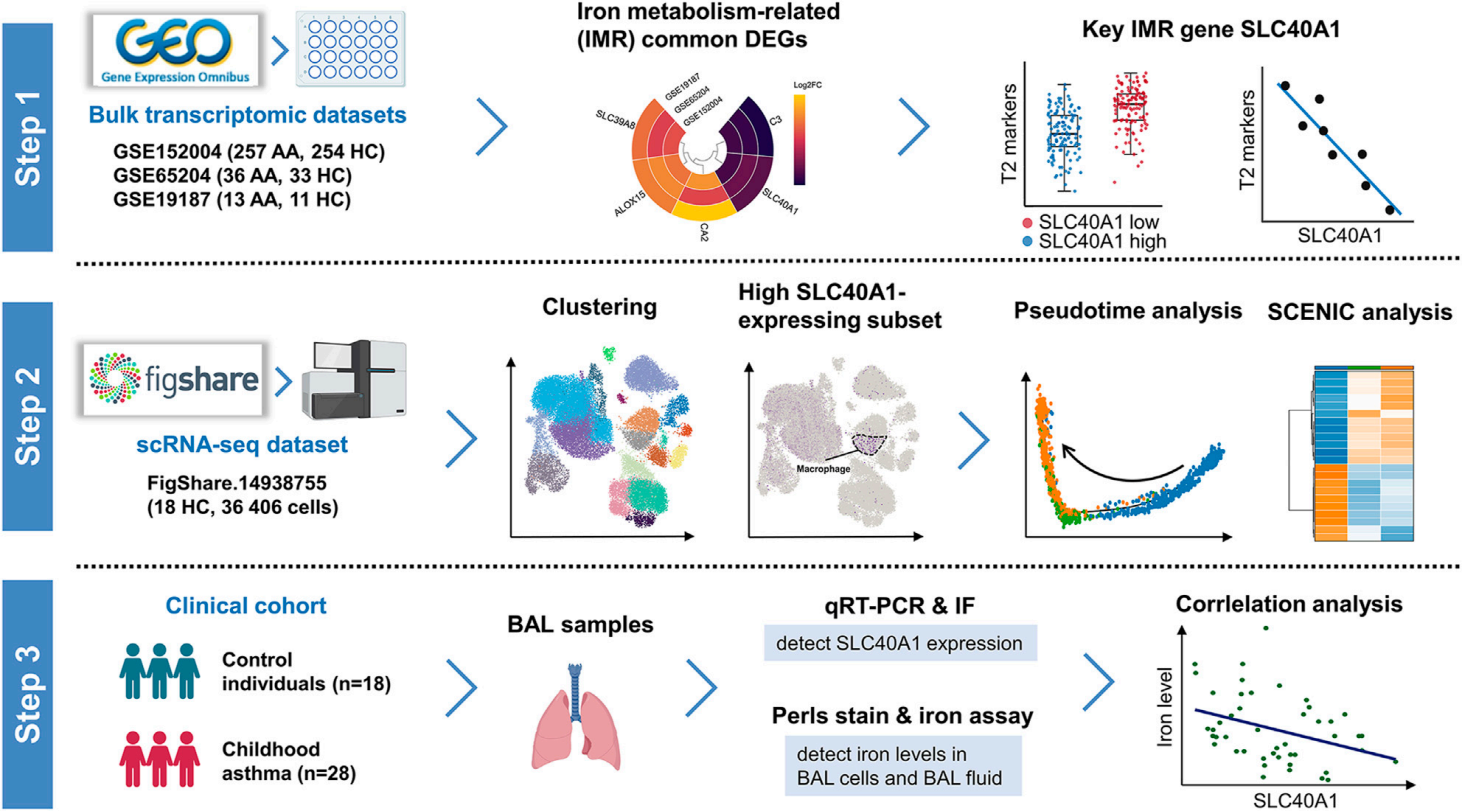
Schematic workflow of study design. Step 1: Identification of SLC40A1 as the key IMR DEG in childhood allergic asthma by bulk transcriptomic analysis. Step 2: Single-cell transcriptomic analysis revealed high SLC40A1-expressing cell subsets. Step 3: Validation of SLC40A1 expression and exploration of its involvement in altered iron levels in the airways of children with allergic asthma. AA, allergic asthma; BAL, bronchoalveolar lavage; DEGs, differentially expressed genes; FC, fold-change; HC, healthy control; IF, immunofluorescence; IMR, iron metabolism-related; qRT-PCR, quantitative reverse transcription-polymerase chain reaction; SCENIC, single-cell regulatory network inference and clustering; scRNA-seq, single-cell RNA sequencing.

The flow chart illustrating the study process and design is presented in Figure 1. Childhood asthma was diagnosed by physicians according to the Global Initiative for Asthma (GINA) (Global Initiative for Asthma, 2021) guidelines based on typical childhood symptoms. Allergy in children was defined as serum-specific IgE positivity to at least one of the tested inhalant allergens or parent-reported history of anaphylaxis (Khoo et al., 2019; Kicic et al., 2020). We collected BAL cells from 31 individual children, including 18 asthmatic patients (eight allergic and 10 non-allergic asthmatics) and 13 controls (children who underwent bronchoscopy based on patient condition with no current respiratory tract infection and no history of allergy, persistent wheezing, or asthma). Collection of BAL from controls and asthmatics was carried out using standard procedures (Experts Group of Pediatric Respiratory Endoscopy, 2018). BALF was gently aspirated and centrifuged at 2,500 rpm for 5 min at 4°C after collection. The BAL cells were collected in phosphate-buffered saline (PBS) and stored at −80°C. Details on subject characteristics are included in Supplementary File 1: Supplementary Table S1.

### Retrieval of IMR genes

Thirty-four IMR gene sets containing 772 IMR genes (Supplementary Table S2) were extracted from the Molecular Signatures Database (MSigDB) (Subramanian et al., 2005) (http://software.broadinstitute.org/gsea/index.jsp).

### Bulk microarray and RNA-seq dataset collection and processing

The mRNA expression profiles of asthmatic children were obtained from the Gene Expression Omnibus (GEO) database (https://www.ncbi.nlm.nih.gov/geo/). The following eligibility criteria were used to include datasets: 1) a minimum of 20 subjects in the dataset, containing both children with allergic asthma and healthy controls; and 2) nasal cells collected by nasal brushing. Three datasets (GSE152004, GSE65204, and GSE19187) met the criteria and were included in the study. The GSE152004 dataset contains 441 asthmatic children and 254 healthy individuals, the GSE65204 dataset contains 36 allergic asthmatic children and 33 healthy individuals, and the GSE19187 dataset contains 13 allergic asthmatic children and 11 healthy individuals.

For GSE19187, gene expression data were acquired by reading raw *CEL* files using the “oligo” R package (v1.58.0) (Carvalho and Irizarry, 2010) with background correction and Robust Multiarray Average (RMA) normalization, after which batch effects were removed using the “sva” package (v3.42.0) (Leek et al., 2012). For GSE65204, microarray raw data were normalized using the “limma” (Ritchie et al., 2015) package (v3.50.0), following background correction using the “normexp” method and quantile normalization and log2 transformation. For the RNA-seq GSE152004 dataset, the raw count matrix of each sample was acquired directly from the dataset.

### Identifying T2-high and T2-low asthmatic children in GSE152004

The T2 immune response is the principal mechanism responsible for allergic asthma (Fainardi et al., 2022). Thus, to identify T2-high and T2-low asthmatic individuals within the GSE152004 dataset, we first performed weighted gene co-expression network analysis (Zhang and Horvath, 2005) (WGCNA) using the “WGCNA” (Langfelder and Horvath, 2008) R package (v4.1.2) to detect T2-high gene-related modules. Pairwise Pearson correlation analysis was conducted for all expressed genes in the dataset and a gene network was constructed based on these correlations. To identify modules of co-regulated genes, hierarchical clustering analysis based on the topological overlap matrix was performed to group genes into modules. We ran the analysis using 17,446 genes and 441 asthma samples. The soft thresholding power was set to 9, deepSplit parameter was set to 2, and minimum module size was set to 30. WGCNA identified 56 modules, with the maroon module found to be enriched in several known T2-high markers, including POSTN, CLCA1, and CST1 (Jackson et al., 2020) (Supplementary Table S3). We used the “factoextra” R package (https://CRAN.R-project.org/package=factoextra) to hierarchically cluster all asthmatic patients based on maroon module expression and used the first split as the basis for assignment to T2-high and T2-low groups. Hierarchical clustering was performed using the “ward.D” method and “Euclidean” distance. Finally, 257 samples were assigned to the T2-high group and 184 samples were assigned to the T2-low group (Supplementary File 1: Supplementary Figure S1). The T2-high asthmatic children and all 254 healthy controls were retained for subsequent analysis.

### Identification and analysis of differentially expressed genes (DEGs)

Differential gene expression analysis between childhood allergic asthmatics and controls was performed for GSE65204 and GSE19187 using the “limma” package and for GSE152004 using the “DESeq2” (Anders and Huber, 2010) package (v1.34.0). Thresholds of *p* < 0.05 and |fold-change| > 1.5 were used to define significant DEGs. Fold-changes, significance, and gene expression patterns of the DEGs were visualized with volcano plots using the “ggplot2” (Ginestet, 2011) package (v3.3.5). To identify common IMR DEGs among the three datasets, the “UpSetR” (Lex et al., 2014) R package (v1.4.0) was used to construct an UpSet diagram, and common IMR DEGs were retained for further analysis.

### Construction of protein-protein interaction (PPI) network and transcriptional regulatory network of common IMR DEGs

The PPI network of common IMR DEGs was constructed using co-expression, pathway, physical interactions, and shared protein domains obtained from the GeneMania database (Warde-Farley et al., 2010).

To recognize potential regulatory transcription factors (TFs) for the common IMR DEGs, we performed enrichment analysis of TF binding motifs (TFBMs) and TFs surrounding the transcription start site (TSS) of the genes using the “RcisTarget” (Aibar et al., 2017) R package (v1.14.0). Significantly enriched TFBMS [normalized enrichment score (NES) > 3.0] were annotated to TFs using the provided annotation database. Finally, the TF-target network was visualized with Cytoscape (v3.8.2) (Shannon et al., 2003).

### Single-cell RNA-seq data acquisition and processing

Single-cell transcriptome data (count matrix) of nasal cells collected from 18 healthy children were obtained from the FigShare repository (https://doi.org/10.6084/m9.figshare.14938755) (Supplementary File 1: Supplementary Table S4). The R package Seurat (Butler et al., 2018) (v4.1.2) was used to preprocess the scRNA-seq data. To ensure high-quality single cells were used for downstream analysis, we filtered out cells with fewer than three genes and cells with more than 15% mitochondrial reads or fewer than 200 genes expressed. The final quality controlled dataset contained 36,406 cells with a mean of 2,390 counts and 2,496 genes per cell (Supplementary File 1: Supplementary Figure S2A).

Raw data were normalized using the “NormalizeData” function, and 2,000 highly variable genes were identified using “FindVariableFeatures.” Subsequently, principal component analysis (PCA) was performed for dimensionality reduction after data scaling. The top 50 principal components were selected to perform downstream analysis. The Uniform Manifold Approximation and Projection (UMAP) algorithm was used for cell visualization.

### Cell clustering and annotation

The “FindClusters” function was used to perform unsupervised cell clustering. To annotate the cell clusters, DEGs for each cell cluster were identified by comparing each cluster to all other clusters with the “FindAllMarkers” function using the default non-parametric Wilcoxon rank sum test with Bonferroni correction. Genes with adjusted *p* < 0.05 were considered as DEGs. The cell subsets were annotated based on the DEGs and known markers from the literature (Chua et al., 2020; Mould et al., 2021; Loske et al., 2022). For subclustering analysis, we applied a similar procedure, including variable gene identification, dimensionality reduction, and clustering identification, to the cluster derived from overall analysis.

### Functional annotation and pathway enrichment analysis

To identify the biological function of DEGs from bulk transcriptional analysis, the common IMR DEGs were subjected to Gene Ontology (GO) (Consortium, 2017) annotation and Kyoto Encyclopedia of Genes and Genomes (KEGG) (Kanehisa et al., 2008) analysis using Metascape (http://metascape.org/gp/index.html), with significant enrichment considered at *p* < 0.01. For the scRNA-seq dataset, cell cluster marker genes were used for KEGG and GO enrichment analysis with “biological process” annotations using the “clusterProfiler” (Yu et al., 2012) R package (v4.2.2), with significant enrichment considered at *p* < 0.05.

### Single-cell regulatory network inference analysis

Single-cell regulatory network inference analysis was performed using the “SCENIC” (Aibar et al., 2017) R package (v1.2.4). First, genes expressed at either very low levels or in too few cells were removed. The GRNBoost2 algorithm was then applied to infer potential transcription factor targets based on expression data with default parameters. Finally, cells were scored for TF regulon activity using the AUCell algorithm. Average regulon activity was compared between clusters in the heatmap using the “pheatmap” R package (v1.0.12) (https://CRAN.R-project.org/package=pheatmap). To identify cell-type-specific regulators for cell clusters of interest, we screened the top five cell-type-specific TFs based on the Regulon Specificity Score (RSS) (Suo et al., 2018) using the “calcRSS” function.

### Single-cell trajectory analysis

We performed pseudotemporal trajectory inference analysis using the “Monocle” (Qiu et al., 2017) R package (v2.22.0). Trajectory analysis was performed with highly variable genes identified by Seurat. The input was created from the raw count matrix of highly variable genes using the “newCellDataSet” function with default parameters. After calculating size factors and estimating dispersions, dimensionality reduction was performed using the “DDRTree” method. Finally, cells were ordered along a pseudotime trajectory with the “orderCells” function. To identify genes that dynamically changed along the trajectory, a DEG test along the cell trajectory was performed using the “differentialGeneTest” function. The expression patterns of representative genes were visualized along the pseudotime using the “pheatmap” R package (v1.0.12).

### Deconvolution of bulk transcriptomic datasets

We performed cell composition estimation using the “BisqueRNA” (Jew et al., 2020) R package (v1.0.5) for the bulk transcriptomic datasets of nasal samples, with the scRNA-seq data as a reference. Default parameters were used during analysis.

### RNA extraction and quantitative reverse-transcription polymerase chain reaction (qRT-PCR)

Total RNA was extracted from human BAL cells using TRIzol reagent (Invitrogen, USA), and purified using a Micro Total RNA Extraction Kit (Tianmo Biotech, China). Total RNA quality was assessed using a NanoDrop 2000 spectrophotometer, and cDNA was synthesized using a PrimeScript RT Kit (TaKaRa, Japan) according to the manufacturer’s instructions. Reactions were carried out in a total volume of 10 μL, including 5 μL of TB Green®Premix Ex Taq™ II (TaKaRa, Japan), 0.2 μL of each specific primer, 2.6 μL of dd H_2_O, and 2 μL of cDNA. Using the 2^−ΔΔCt^ method, the relative expression levels of target genes were calculated. GAPDH was used as an internal reference. Specific primers for each gene and cycling conditions are provided in Supplementary File 1: Supplementary Table S5.

### Perls stain

In order to observe the distribution of iron in BAL cells, Perls Prussian blue staining was performed according to the manufacturer’s kit protocol (Prussian Blue Iron Stain Kit, Solarbio, China).

### Iron assay

Iron concentrations in the BALF were measured via the chromogen method with an Iron Assay Kit (MAK025, Sigma-Aldrich, United States) according to the manufacturer’s instructions (Wang et al., 2019).

### Immunofluorescence

BAL cells were washed twice with sterile PBS and fixed with 4% paraformaldehyde (FA) for 10 min. FA fixed cells were washed three times with PBS and then blocked in 5% bovine serum albumin (BSA) for 30 min. Primary antibody incubations were performed overnight at 4°C. Secondary antibody incubations were performed for 1 h at room temperature followed by washing with PBS. CD68 was detected with FITC-conjugated mouse anti-CD68 antibody [eBioY1/82A (Y1/82A), eBioscience, United States] diluted 1/200 times, FPN with rabbit anti-FPN (PA5-22993, Invitrogen, United States) diluted 1/500 times, TFR1 with rabbit anti-TFR1 (R25971, ZENBIO, China) diluted 1/100 times, DMT1 with rabbit anti-DMT1 (20507-1-AP, Proteintech, China) diluted 1/100 times. As secondary antibody, CY3-conjugated goat anti-rabbit IgG (A0516, Beyotime, China) diluted 1/200 was used. Samples were observed with a fluorescence microscope (BX53, Olympus, Japan).

### Statistical analysis

Continuous data are expressed as mean ± standard deviation (SD). All statistical analyses were conducted using R software (v4.1.2; https://www.r-project.org/). Wilcoxon rank-sum test was used to compare gene expression levels (Figures 3D–F, 4E, 6E) and inferred cellular composition between groups (Figure 5G). Multiple group comparisons were performed using the Kruskal–Wallis test with Dunn’s multiple comparisons (Figures 6A,C, Supplementary File 1: Supplementary Figures S5A, B). For correlation analyses (Figures 3G–I, 6F), correlation coefficients (r) and *p* values were acquired by Pearson correlation test. For all the above statistical analyses, a *p*-value of <0.05 was considered statistically significant. To determine the power of genes of interest for distinguishing childhood allergic asthmatics from controls, receiver operating characteristic (ROC) curves and corresponding area under the ROC curves (AUC) were calculated for logistic regression analyses incorporating all five common iron metabolism-related (IMR) DEGs across all datasets (Supplementary File 1: Supplementary Figures S3D–F) or mRNA expression value of SLC40A1 relative to GAPDH (Figure 6G). Statistic methods and associated threshold used for other analysis are detailed in the specific method sections.

## Results

### Identification of IMR DEGs in childhood allergic asthma

**FIGURE 2:**
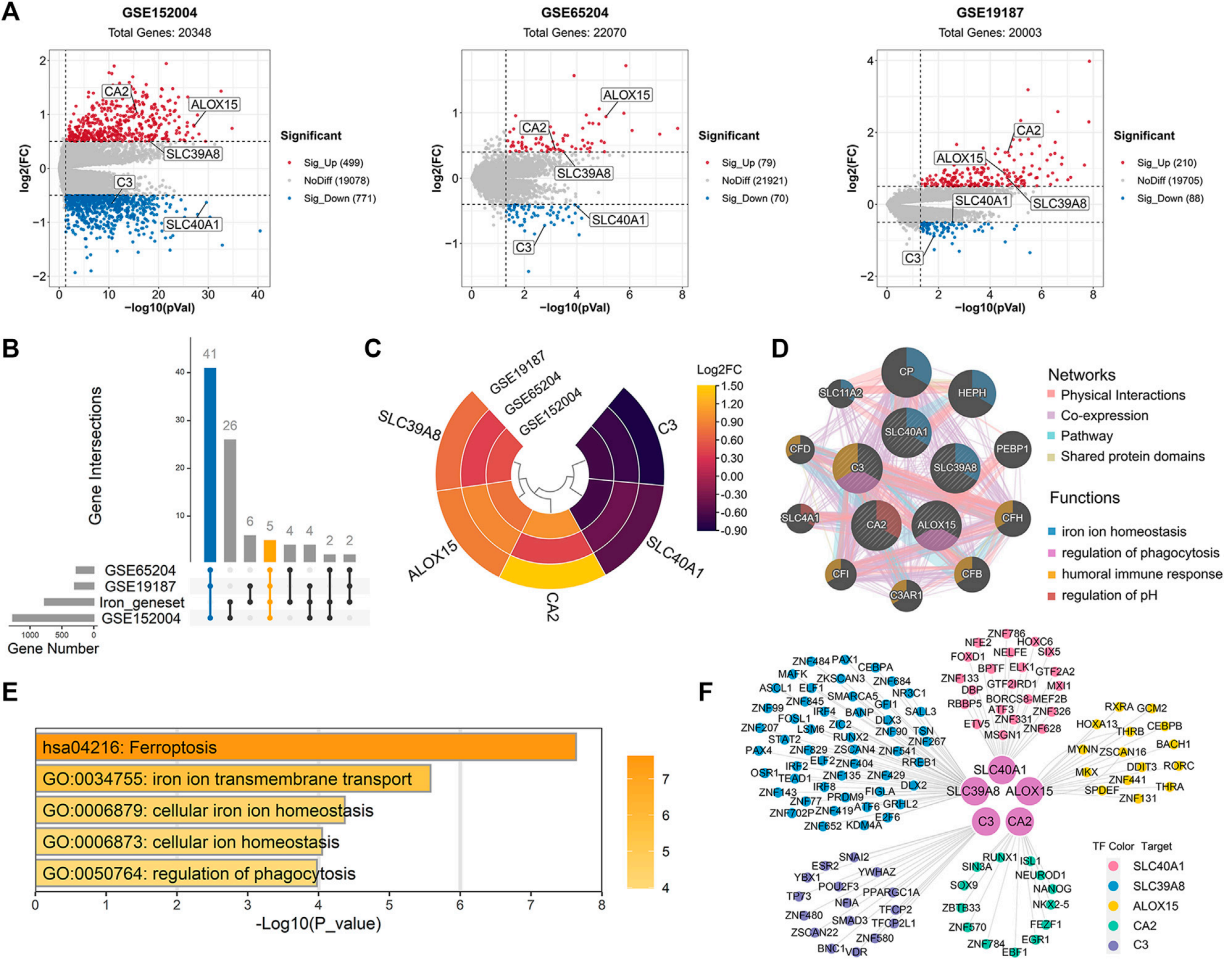
Identification and enrichment analysis of dysregulated IMR genes in childhood allergic asthma. **(A)** Volcano plots of DEGs between healthy controls and children with allergic asthma. **(B)** UpSet diagram showing overlapping DEGs and overlapping IMR DEGs among the three datasets. Datasets shared five common IMR DEGs. **(C)** Heatmap of common IMR DEGs derived from integrated analysis. Each circle represents one dataset, and each sector represents one gene. **(D)** Protein-protein interaction network for common IMR DEGs using GeneMANIA database. Each node represents a protein (labelled with gene name); node color represents possible function of respective protein; and line color of connections represents different interaction type. **(E)** Significantly enriched GO terms and KEGG pathways of common IMR DEGs using Metascape. **(F)** TF-gene regulatory network of common IMR DEGs constructed using “RcisTarget” R package. Hub nodes represent five common IMR DEGs; outer nodes represent TFs; and each color indicates enriched TFs for common IMR DEG. DEGs, differentially expressed genes; FC, fold-change; GO, gene ontology; KEGG, kyoto encyclopedia of genes and genomes; IMR, iron metabolism-related; TF, transcription factor.

Compared with healthy controls, we initially identified 1,270 DEGs (499 upregulated and 771 downregulated), 149 DEGs (79 upregulated and 70 downregulated), and 298 DEGs (210 upregulated and 88 downregulated) in childhood allergic asthma in the GSE152004, GSE65204, and GSE19187 bulk transcriptomic datasets, respectively (Figure 2A). The UpSet diagram in Figure 2B shows the number of overlapping DEGs among the three datasets and the overlapping IMR DEGs among the datasets. There were 41 common DEGs and five common IMR DEGs, including SLC40A1, SLC39A8, ALOX15, CA2, and C3, across the datasets. Among them, SLC39A8, ALOX15, and CA2 showed higher expression, while SLC40A1 and C3 exhibited lower expression in patients with allergic asthma (Figure 2C). These five common IMR DEGs were retained for subsequent analysis. Detailed information on the above datasets is shown in Supplementary File 1: Supplementary Table S4.

### Functional annotation and construction of transcriptional regulatory network of IMR DEGs

To investigate the interactions between proteins encoded by common IMR DEGs and other proteins, a PPI network was constructed. Results showed that five proteins encoded by the common IMR DEGs were surrounded by 10 potentially interacting proteins (Figure 2D). Notably, SLC40A1 and SLC39A8 and their interacting proteins are involved in the regulation of iron homeostasis (Figure 2D). To further investigate the latent biological behaviors of the common IMR DEGs, GO enrichment analysis indicated that these genes were mainly involved in iron ion transmembrane transport, cellular iron ion homeostasis, and regulation of phagocytosis (Figure 2E). Moreover, the ferroptosis pathway was the major biological pathway involved (Figure 2E). Ferroptosis is a newly identified form of cell death characterized by cellular iron accumulation and lipid peroxidation (Jiang et al., 2021; Tang et al., 2021). Excess cellular iron due to dysregulation of IMR-molecules can promote ferroptosis (Jiang et al., 2021). Together, these results suggest that disturbance in iron metabolism may be involved in the pathogenesis of childhood allergic asthma.

Next, we used TFBM enrichment analysis to identify potential upstream TFs for the common IMR DEGs. The TF-gene interaction network was constructed, which included five genes and 118 TFs (Figure 2F). SLC40A1, SLC39A8, ALOX15, C3, and CA2 were regulated by 21, 51, 51, 17, and 16 TFs, respectively. The enriched TFs are shown in Supplementary Table S6.

### SLC40A1 was closely related to T2 airway inflammatory markers

**FIGURE 3:**
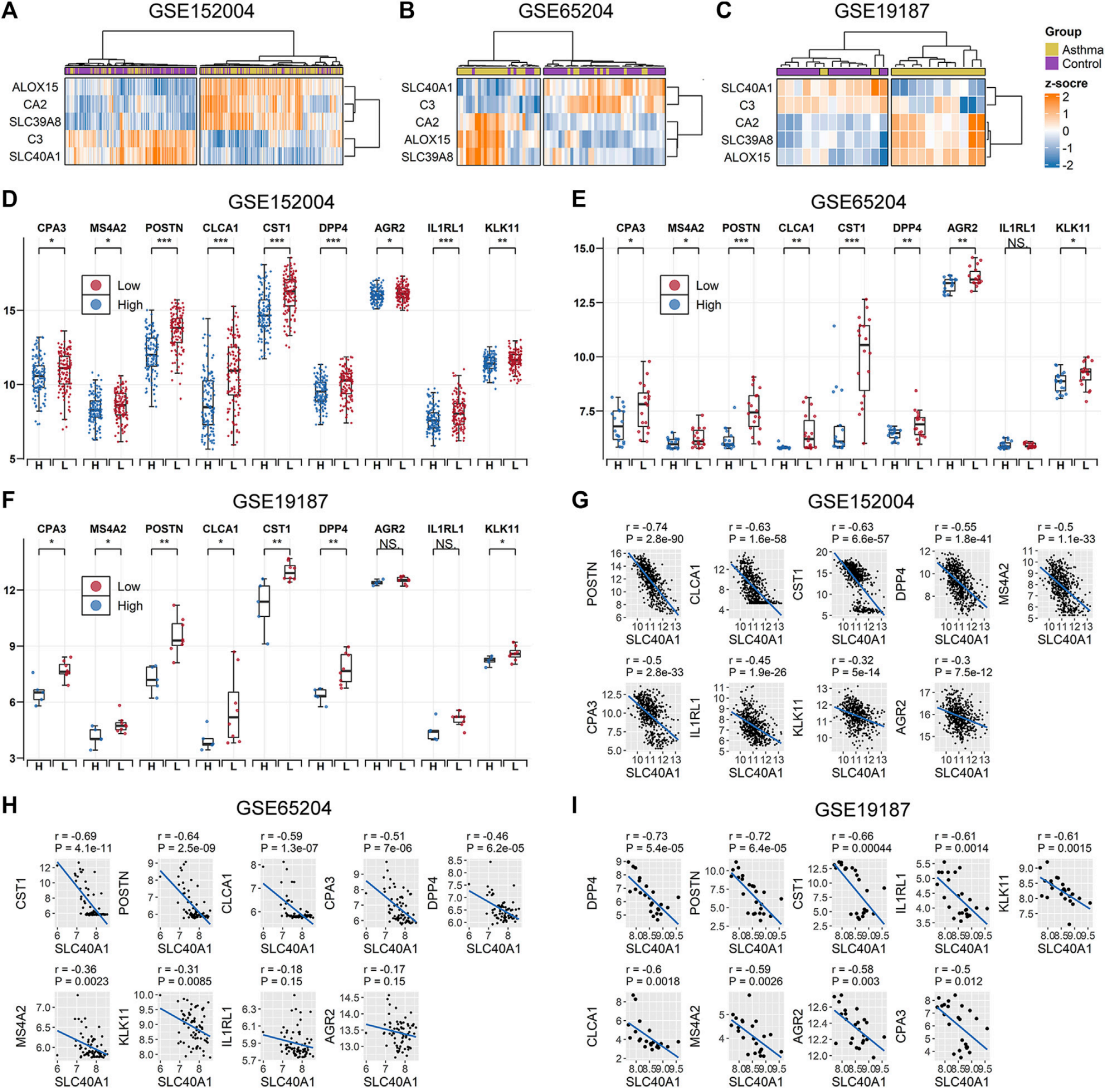
Association between SLC40A1 and type 2 airway inflammatory markers. Hierarchical clustering of all patients using common IMR DEGs distinguished allergic asthmatic patients from controls in **(A)** GSE152004, **(B)** GSE65024, and **(C)** GSE19187 datasets. Hierarchical clustering was performed using the “ward.D” method and “Euclidean” distance. Comparison of gene expression levels of nine T2 airway inflammatory markers between SLC40A1 high-expression group **(H)** and SLC40A1 low-expression group (L) of all asthmatic patients in **(D)** GSE152004, **(E)** GSE65024, and **(F)** GSE19187. Statistical significance was assessed using Wilcoxon rank-sum test. Asterisks indicated *p* values for SLC40A1 high-expression versus SLC40A1 low-expression. *p < 0.05, **p < 0.01, ***p < 0.001. Scatter plots of SLC40A1 vs. type 2 airway inflammatory marker expression in **(G)** GSE152004, **(H)** GSE65024, and **(I)** GSE19187. Correlation coefficients (r) and *p*-values were obtained by Pearson correlation analysis. DEGs, differentially expressed genes; NS, no significance; IMR, iron metabolism-related.

We further explored whether the common IMR DEGs could be distinguished between children with allergic asthma and healthy subjects. Using the five common IMR DEGs, we performed independent unsupervised hierarchical clustering of all patients derived from the different datasets. Heatmap visualization of the five common IMR DEGs indicated that 1) hierarchical clustering grouped samples into two major clusters and 2) hierarchical clustering based on expression profiles of common IMR DEGs clearly distinguished patients from controls in GSE65204 and GSE19187 datasets (Figures 3A–C). Although PCA based on the five common IMR DEGs well separated patients from controls in GSE19187, but was unable to clearly distinguish asthmatics from controls in GSE152004 and GSE65204 (Supplementary File 1: Supplementary Figures S3A–C).To evaluate the power of common IMR DEGs to distinguish childhood allergic asthmatics from healthy individuals, we measured the AUC values derived from logistic regression analyses incorporating all five common IMR DEGs across the datasets. The ROC analyses showed that all five IMR DEGs displayed moderate-high ability (AUC from 0.81–0.99) to distinguish allergic asthmatic children from healthy controls in the three datasets (Supplementary File 1: Supplementary Figures S3D–F)**.**

Ferroportin (encoded by SLC40A1) is the only recognized mammalian cellular iron exporter (Neves et al., 2019; Zhang et al., 2019). Given its importance in cellular iron homeostasis, we explored whether SLC40A1 plays a potential role in childhood allergic asthma. We stratified all asthmatic patients across the datasets into two groups based on median SLC40A1 expression and compared the gene expression levels of nine markers of T2 airway inflammation identified in previous studies (Jackson et al., 2020; Kicic et al., 2020). Interestingly, genes related to T2 airway inflammation showed an overall elevated trend in patients with low SLC40A1 expression in each dataset (Figures 3D–F). We also observed negative correlations between the expression levels of SLC40A1 and T2 inflammatory markers (Figures 3G–I). Taken together, these results implicate the potential role of SLC40A1 in T2 airway inflammation in childhood allergic asthma.

### Single-cell transcriptomic analysis revealed high SLC40A1-expressing cell subpopulations within airway

**FIGURE 4:**
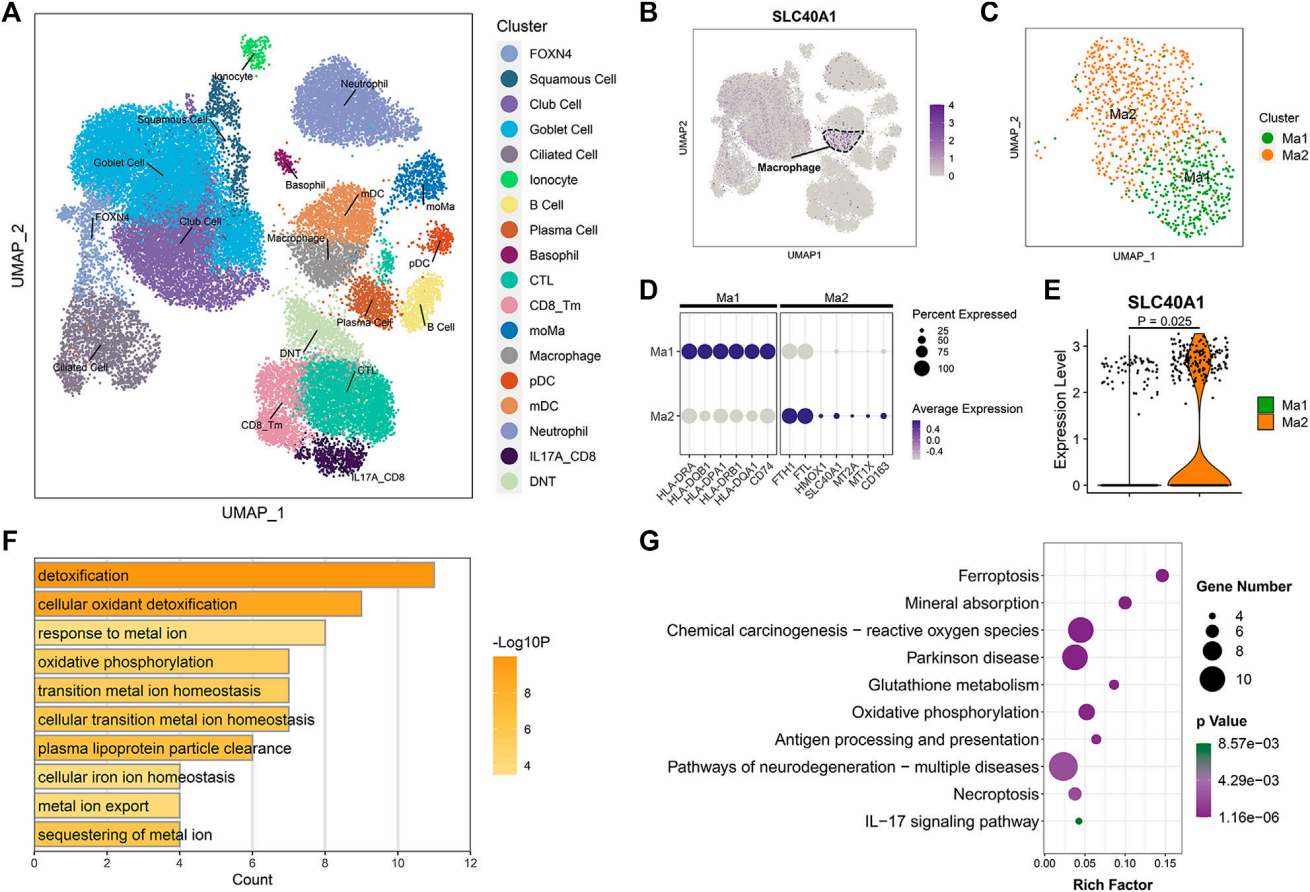
Single-cell transcriptomic analysis revealed high SLC40A1-expressing cell subpopulations within airway. **(A)** Overall cell-type composition of 36,406 cells from 18 healthy children visualized using UMAP. CD8_Tm, memory CD8^+^ T cells; CTL, cytotoxic T cells; DNT, double-negative T cells; FOXN4, FOXN4^+^ cells; IL-17A_CD8, IL-17A-expressing CD8^+^ T cells; mDC, myeloid dendritic cells; moMa, monocyte-derived macrophages; pDCs, plasmacytoid dendritic cells. **(B)** Feature plot of SLC40A1 expression in all cell populations. **(C)** Subclustering of macrophages, showing two distinct subpopulations (Ma1 and Ma2). **(D)** Dot plots of scaled expression levels of canonical markers used to identify each macrophage subtype. Color key from gray to purple indicates low to high expression levels. Dot size indicates percentage of cells that expressed genes. **(E)** Violin plot of SLC40A1 expression for macrophage subsets. Statistical significance was assessed using Wilcoxon rank-sum test. **(F)** Enriched GO terms for DEGs in Ma2 macrophage subset. **(G)** KEGG enrichment analysis of DEGs in Ma2 macrophage subset. GO, gene ontology; KEGG, kyoto encyclopedia of genes and genomes; UMAP, uniform manifold approximation and projection.

To identify which specific cell types within the airway express SLC40A1, we employed scRNA-seq data using cells from the upper airways of 18 healthy children (Loske et al., 2022). After stringent quality control, 36,406 cells were subjected to further analysis. Unsupervised clustering by UMAP identified 18 cell subtypes (Figure 4A) based on representative marker genes from previous studies (Chua et al., 2020; Loske et al., 2022). The cell populations identified by cell lineage-specific marker gene expression are shown in Supplementary File 1: Supplementary Figures S2B, C. We investigated SLC40A1 expression in each cell subset and found that macrophages expressed the highest level of SLC40A1 (Figure 4B). Thus, the following analyses focused on the macrophage subpopulation.

We re-clustered the macrophages to further explore the distribution of SLC40A1 expression. Macrophages were re-clustered into two distinct subpopulations (Ma1 and Ma2) (Figure 4C). The Ma1 cluster expressed high levels of antigen processing and presentation-associated genes, such as HLA-DRA, HLA-DQB1, and HLA-DPA1 (Figure 4D; Supplementary Table S7). The Ma2 cluster showed high expression of IMR genes SLC40A1, HMOX1, FTH1, and FTL, metallothionein genes MT2A and MT1X, and resident macrophage marker CD163 (Figures 4D, E; Supplementary Table S7). GO analysis of DEGs in cluster Ma2 indicated enrichment in metal ion homeostasis and IMR terms (Figure 4F). Furthermore, pathway analysis revealed that Ma2 was enriched in multiple pathways, including ferroptosis and mineral absorption (Figure 4G). Of note, several recent scRNA-seq studies identified an alveolar macrophage cluster that expresses metallothionein (Mould et al., 2021; Sauler et al., 2022). In our analysis, we found that a macrophage subpopulation in the upper airway exhibited concurrent high expression of metallothionein and IMR genes. Taken together, our findings revealed high expression of SLC40A1 in the Ma2 macrophage subset. However, the specific role of this subpopulation in physiological and pathophysiological states requires further exploration.

### Potential biological role of SLC40A1 based on single-cell trajectory and transcriptional regulatory network analyses

**FIGURE 5:**
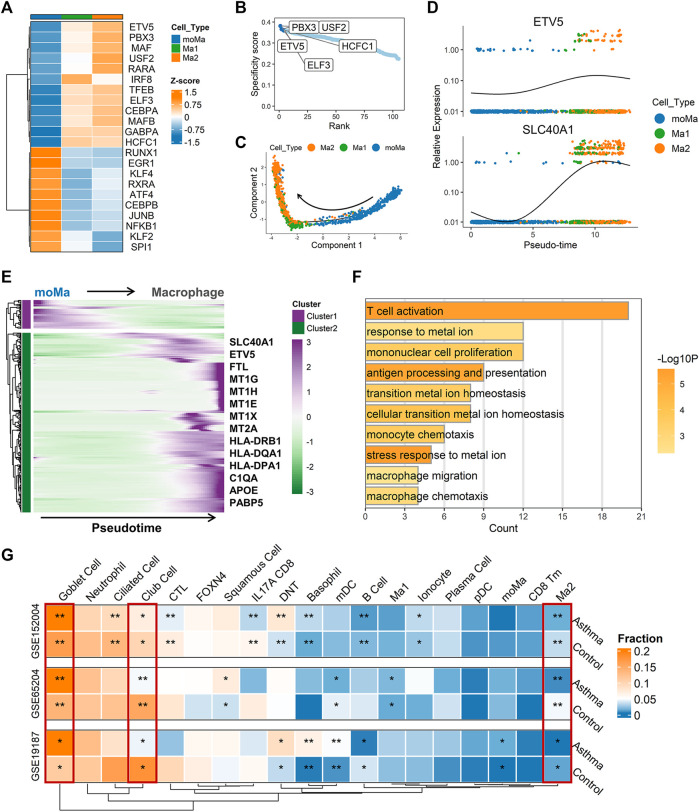
Single-cell trajectory and transcriptional regulatory network analysis revealed potential biological role of SLC40A1. **(A)** Heatmap of transcription factor (TF) activity (rows) in macrophages (subclusters Ma1 and Ma2) and moMa (columns), as identified by SCENIC. **(B)** Ranks of TFs in macrophage Ma2 subset based on regulon specificity scores, with five most transcriptionally active TFs screened. **(C)** Monocle pseudotime trajectory analysis of moMa and macrophages (subclusters Ma1 and Ma2), showing a major trajectory of moMa differentiation into macrophages. Direction of arrow indicates pseudotime direction. **(D)** Feature plot showing the expression patterns of ETV5 and SLC40A1 along the pseudotime cell trajectory. **(E)** Gene expression dynamics along pseudotime trajectory. Genes clustered into two gene sets, each characterized by specific expression profiles. Genes in cluster 2 exhibited increased expression along pseudotime, as depicted by a selection of characteristic genes. Color bars from green to purple represent *Z* score. **(F)** Enriched GO terms for genes in cluster 2. **(G)** Heatmap of proportion of major cell subtypes within airways of allergic asthmatic patients and healthy controls in each dataset predicted from deconvolution analysis. Statistical significance was assessed using Wilcoxon rank-sum test. Asterisks indicated *p* values for childhood allergic asthma versus control. *p < 0.05, **p < 0.01. GO, gene ontology; moMa, monocyte-derived macrophages; SCENIC, single-cell regulatory network inference and clustering.

Once monocytes are released into circulation from bone marrow, they can migrate to peripheral tissue and differentiate into tissue-resident macrophages under homeostasis and inflammation (Guilliams et al., 2018). To clarify possible developmental connections between macrophage subsets and monocyte-derived macrophages (moMa), we performed pseudotime analysis using Monocle, which ordered cells along the pseudotime trajectory based on gene expression patterns (Figure 5C). Results showed that moMa, Ma1, and Ma2 were primarily positioned at the start, middle, and end of the pseudotime trajectory, respectively (Figure 5C). Indeed, single-cell regulatory network analysis indicated increasing TF activity associated with macrophage maturation (Kurotaki et al., 2017; Zhao et al., 2018), with MAFB and CEBPA moving from moMa to Ma1 to Ma2 (Figure 5A). These results suggest that moMa may exist along a continuum of maturation towards mature macrophages, while Ma2 macrophage are in the late stage of maturation.

As the Ma2 subpopulation showed the highest level of SLC40A1 expression (Figure 4E), we explored the potential upstream regulators of Ma2. Based on RSS, we identified the top five TFs (Figure 5B) with major transcriptional regulatory roles in the Ma2 subset, including ETV5. Notably, ETV5 was also shown to be an upstream TF of SLC40A1 based on the bulk transcriptional regulatory network (Figure 2F). Furthermore, ETV5 activity progressively increased during the differentiation of moMa into macrophages (Figure 5A), and thus we investigated the potential roles of ETV5 and SLC40A1 in this process using pseudotime analysis. During moMa to macrophage maturation, ETV5 expression increased with pseudotime and was co-expressed with SLC40A1 (Figure 5D), suggesting that ETV5, together with SLC40A1, may be involved in the differentiation process. Furthermore, we explored dynamic transcriptional changes in gene expression during differentiation from moMa into macrophages, resulting in two clusters with different time-dependent expression patterns (Figure 5E). Cluster 2 consisted of 314 genes (Supplementary Table S8), including SLC40A1 and ETV5, which showed increased expression with pseudotime, underscoring their possible roles in moMa to macrophage differentiation. The biological functions of genes in cluster 2 were also assessed using GO analysis. Results indicated that these genes were enriched in several pathways involved in metal ion metabolism, antigen processing and presentation, and T cell activation (Figure 5F). These findings suggest that SLC40A1 and ETV5 may be involved in the differentiation of moMa into macrophages.

### Deconvolution of bulk transcriptomic data to infer changes in cell composition within airways of children with allergic asthma

Asthma is a heterogeneous disease involving complex interactions of multiple cell types. However, comprehensive investigations of changes in the airway microenvironment of children with allergic asthma are limited. Deconvolution of bulk tissue RNA to infer cellular composition has the potential to contribute to a better understanding of the pathogenesis of multiple diseases (Huang et al., 2022). Thus, we performed gene deconvolution of the three bulk transcriptomic datasets to deduce changes in cell type frequency within the airways of children with allergic asthma.

Compared with the healthy controls, a higher proportion of goblet cells and a lower proportion of club cells were observed in the asthmatic airways across all datasets (Figure 5G). Interestingly, allergic asthmatics also showed a lower fraction of SLC40A1-expressing Ma2 macrophages (Figure 5G). The significance of this cell subpopulation, which is implicated in iron metabolism, deserves further exploration in childhood allergic asthma. However, differences in several cell fractions (e.g., B cells and basophils) between groups were not completely consistent across the datasets. Thus, single-cell RNA-seq studies on childhood asthma are necessary to further explore the cellular heterogeneity within the complex asthmatic airway microenvironment.

### Effects of SLC40A1 on altered iron levels in airways of children with allergic asthma

**FIGURE 6:**
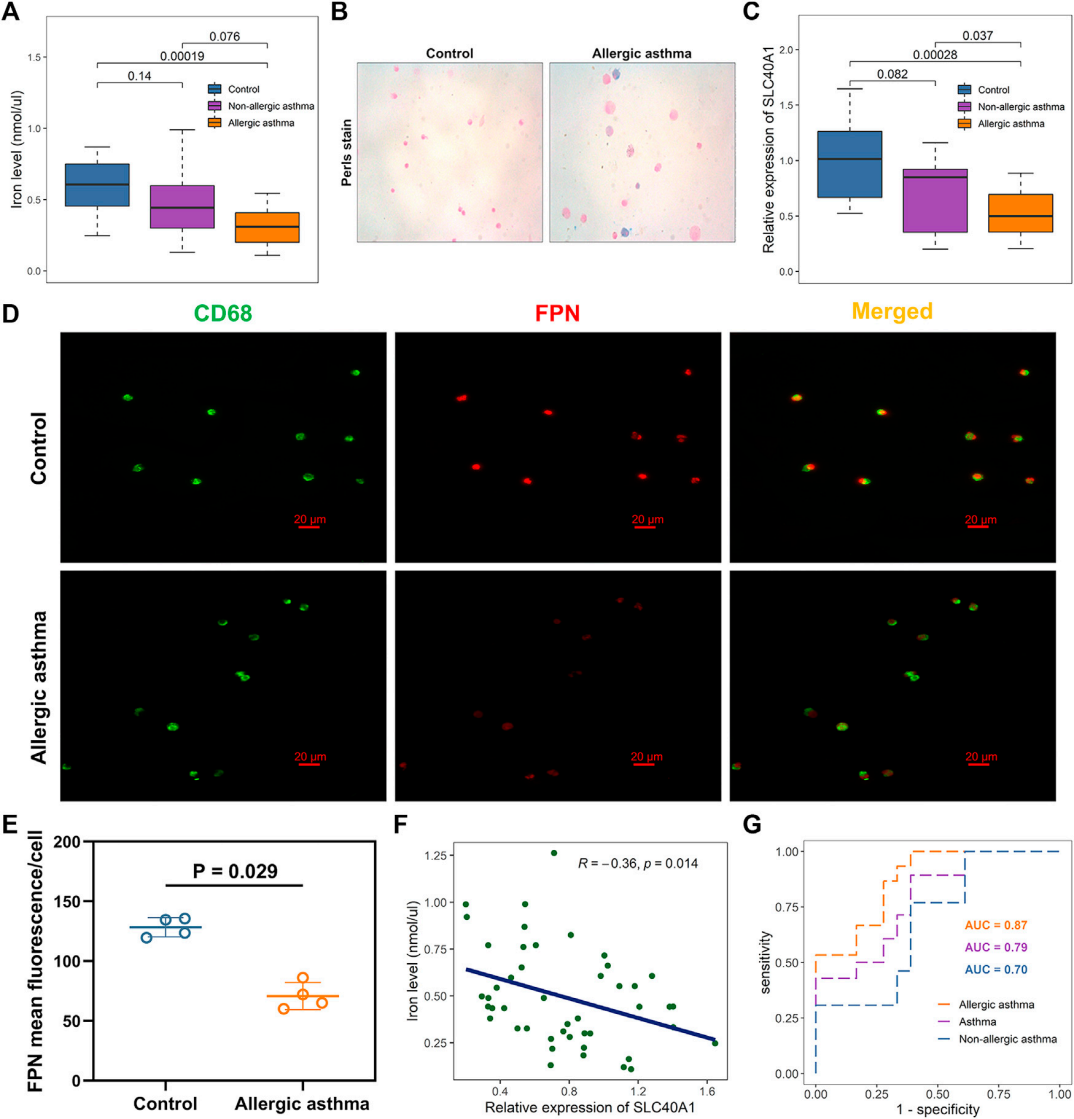
Effects of SLC40A1 on altered iron levels in airways of children with allergic asthma. **(A)** Boxplot showing differences in BALF iron levels among groups, as determined by iron assay. Statistical significance was assessed using Kruskal–Wallis test with Dunn’s multiple comparisons. **(B)** Perls iron stain of BAL cells from control individuals and childhood allergic asthmatics (blue stain represents iron positive areas). **(C)** Boxplot showing mRNA expression levels of SLC40A1 among groups. mRNA expression of SLC40A1 was measured by qRT-PCR. Statistical significance was assessed using Kruskal–Wallis test with Dunn’s multiple comparisons. **(D)** Representative immunofluorescent images of BAL cells stained with anti-CD68 antibody and anti-FPN antibody. **(E)** FPN mean fluorescence/cell per group. Statistical significance was assessed using Wilcoxon rank-sum test. **(F)** Scatter plot of relative SLC40A1 expression vs. BALF iron levels. Correlation coefficients (r) and *p*-values were obtained by Pearson correlation analysis. **(G)** ROC curve evaluating diagnostic utility of SLC40A1 for childhood asthma. BAL, bronchoalveolar lavage, BALF, bronchoalveolar lavage fluid; AUC, area under ROC curve; qRT-PCR, quantitative reverse-transcription polymerase chain reaction; ROC, receiver operating characteristic.

Analysis showed that SLC40A1 exhibited much lower expression in the nasal samples of patients with allergic asthma (Figure 2C). BAL can provide direct and useful information about local airway inflammation (Fainardi et al., 2022). Thus, to investigate potential iron metabolism disorder in the airways of children with allergic asthma, we examined iron levels and SLC40A1 expression using qRT-PCR analysis of BAL samples from 18 controls and 28 asthmatic children. We also assessed iron distribution in BAL cells using Perls iron staining. Results indicated that iron levels in BALF were significantly lower in children with allergic asthma than in controls (*p* = 0.00019) (Figure 6A). In contrast, children with allergic asthma showed significant iron accumulation in BAL cells compared to controls (Figure 6B). Together these results suggest that decreased iron levels in the airway, but increased iron accumulation in BAL cells in childhood allergic asthma. Furthermore, SLC40A1 mRNA expression was markedly decreased in allergic asthmatic children compared to controls (*p* = 0.00028), and there was no statistical significance between controls and non-allergic asthmatics (Figure 6C). Single-cell transcriptomic analysis suggested that macrophages expressed the highest level of SLC40A1 (Figure 4B). To clarify the protein expression of SLC40A1 in BAL macrophages, immunofluorescence staining of BAL cells was performed in which macrophages were stained by anti-CD68. Results showed that the expression of FPN was mainly co-localized with CD68 staining in BAL cells (Figure 6D). Furthermore, fluorescence intensity quantification showed lower levels of FPN in allergic asthmatic samples than in control samples (Figure 6E).

Most cells acquire iron by importing transferrin-bound iron from the extracellular environment via TFR1 (Neves et al., 2019). As another cellular iron importer, DMT1 is responsible for transporting dietary non-heme iron through the cellular membrane (Zhang et al., 2019). Here, we further investigated whether the decreased iron levels in BALF were due to elevated expression of TFR1 or DMT1. However, we found no differences in the TFR1 and DMT1 mRNA expression levels between the asthmatic children and controls (Supplementary File 1: Supplementary Figures S5A, B). Immunofluorescent staining also showed similar TFR1 and DMT1 staining between the groups (Supplementary File 1: Supplementary Figures S4A, B).

Pearson correlation analysis showed a negative association between SLC40A1 expression and iron levels (Figure 6F). Furthermore, SLC40A1 displayed moderate discriminatory power in distinguishing overall asthma [area under the ROC (AUC) = 0.79] and non-allergic asthma (AUC = 0.70) from controls, and high discriminatory power to distinguish between allergic asthma and controls (AUC = 0.87) (Figure 6G). Taken together, these results suggest that lower SLC40A1 expression may be correlated with reduced iron levels in the airways of children with allergic asthma.

## Discussion

Despite mounting evidence implicating iron metabolism disorder in childhood asthma pathogenesis, the underlying mechanism remains unclear. In the current study, we identified common aberrantly expressed IMR genes in childhood allergic asthma via integrative analysis of three bulk transcriptomic datasets. Among these genes, SLC40A1 was strongly correlated with markers of T2 airway inflammation. Subsequent single-cell transcriptomic analysis identified a distinct subset of macrophages driving SLC40A1 expression and revealed the potential biological role of SLC40A1. Reduced SLC40A1 expression may be associated with declined iron levels in the airways of children with allergic asthma.

We performed integrative analysis of three bulk gene expression profiles and identified five common IMR DEGs in childhood allergic asthma, including C3 and ALOX15, which are implicated in allergic asthma pathogenesis (Hasegawa et al., 2004; Yang et al., 2012; Nagasaki et al., 2022). Complement component C3 plays a central role in the complement system and is required for the three complement-activating pathways. A Japanese study of 864 asthmatic patients and controls identified a single-nucleotide polymorphism (SNP) in the C3 gene associated with childhood allergic asthma (Hasegawa et al., 2004). Another adult study reported decreased expression of C3 in the bronchial epithelium of allergic asthmatic patients compared with normal controls (Yang et al., 2012), similar to our results. ALOX15 is a member of the lipoxygenase (LOX) family, which catalyzes the peroxidation of polyunsaturated fatty acids (Çolakoğlu et al., 2018). Under T2 conditions (IL-13 stimulation), upregulation of ALOX15 in human airway epithelial cells has been shown to induce hydroperoxyl-phospholipid generation and lower intracellular glutathione (GSH) (Nagasaki et al., 2022). Lowering GSH by inhibiting SLC7A11 enhances periostin (POSTN, a T2 inflammatory marker) protein expression and increases susceptibility to ferroptotic death (Nagasaki et al., 2022). SLC40A1 encodes ferroportin, the only known cellular iron exporter in mammals (Neves et al., 2019; Zhang et al., 2019). SLC39A8 is widely expressed in tissues and encodes the ZIP8 protein, which is a multi-functional membrane transporter that influxes essential metals such as iron, zinc, and manganese (Liu et al., 2018). Carbonic anhydrase II (CA2) plays physiological roles in erythrocytes, including ion secretion, CO_2_ transport, and pH regulation (Supuran, 2008). To date, however, few studies have reported on the relationship between SLC40A1, SLC39A8, CA2, and asthma, or examined the functional roles of the five IMR genes in iron homeostasis imbalance. Thus, the identified IMR genes warrant further investigation, especially their roles in asthma-related dysregulation of iron metabolism, which may help elucidate novel mechanisms related to childhood allergic asthma.

Interestingly, we found that allergic asthmatic children with lower SLC40A1 expression exhibited higher T2 airway inflammatory gene expression. Identification of potentially valuable biomarkers in asthma may aid risk stratification and management of patients (Kuruvilla et al., 2019). Several T2 airway inflammatory biomarkers, including POSTN, CPA3, IL1RL1, KLK11, and DPP4, which may facilitate evaluation of disease severity and predictive response to therapy, have been identified in adult asthmatics (Zissler et al., 2016; Mogensen et al., 2020; Winter et al., 2021). However, existing data on the pediatric asthma population are limited. Given the high correlation between SLC40A1 and T2 airway inflammatory genes, we hypothesized that stratification of children with allergic asthma based on SLC40A1 expression may help predict treatment response and disease severity. Nevertheless, we could not draw further conclusions due to the lack of individual patient data. Thus, further studies on SLC40A1 and other possible biomarkers of childhood allergic asthma are warranted.

Based on scRNA-seq data, SLC40A1 was highly expressed in the Ma2 macrophage subset, which expressed genes related to iron metabolism and metallothionein. Functional annotation analysis revealed that these cells were enriched in biological processes related to iron metabolism and metal ion homeostasis. In terms of transcriptional phenotype and potential function, these cells may correspond to a novel associated alveolar macrophage subpopulation (Mould et al., 2021; Sauler et al., 2022). However, little is known about the effects of accumulation or scarcity of these cells in the pathogenesis of lung disease (Sauler et al., 2022). The specific role of the SLC40A1-expressing macrophage subset under physiological and pathophysiological states deserves further exploration.

Based on pseudotime and SCENIC analysis, our study also indicated that SLC40A1 and ETV5 may be involved in the differentiation of moMa into macrophages. A previous study found that ETV5 was significantly upregulated during differentiation of primary human monocytes into macrophages (Liu et al., 2008). Further functional studies are necessary to confirm and characterize the roles of ETV5 and SLC40A1 in moMa to macrophage differentiation. Even under homeostatic conditions, bone marrow-derived monocytes can differentiate into resident tissue macrophages, which may be accelerated by inflammation (van de Laar et al., 2016; Gundra et al., 2017). However, the mechanisms that enable the conversion of monocytes into tissue-resident macrophages are unknown. Previous research has indicated that moMa can differentiate into tissue-resident peritoneal macrophages in *Schistosoma mansoni*-infected mice, but failure to convert moMa into a tissue-resident phenotype is associated with dysregulated inflammation and increased mortality (Gundra et al., 2017). A recent study also revealed that activation of the IL33-ST2 axis can facilitate moMa differentiation into resident alveolar macrophages and promote bronchial epithelial repair in a mouse model of naphthalene-induced bronchiolar epithelial injury (Dagher et al., 2020). These studies suggest that the conversion of moMa to macrophages with a resident phenotype contributes to tissue homeostasis. Thus, we hypothesize that conversion of moMa into macrophages with a resident phenotype is disrupted in children with allergic airway inflammation, with the potential involvement of SLC40A1 and ETV5. However, further studies are required to confirm this conjecture.

Using deconvolution analysis, we found that the proportion of goblet cells was increased, and the promotion of club cells was decreased in allergic asthmatic airways. The airway epithelium is the first line of defense against pathogenic factors. Prominent goblet cell hyperplasia is a typical pathological feature of asthma (Carlier et al., 2021). Moreover, airway epithelial injury and dysregulated epithelial barrier function play key roles in the development and progression of asthma (Heijink et al., 2020; Carlier et al., 2021). In response to injury, epithelial regeneration is largely driven by multipotent progenitor stem cells of the bronchial conducting airways, specifically a subset of club progenitor stem cells (Hong et al., 2001). Consistent with our findings, a previous scRNA-seq study revealed that goblet cells are increased, and club cells are reduced in the airways of adult asthmatic patients compared to healthy controls (Vieira Braga et al., 2019). Intriguingly, we found that the Ma2 macrophage subset was decreased in the airways of asthmatic children. Recent mouse model studies have revealed an important dichotomy, in which resident macrophages primarily maintain lung homeostasis by suppressing inflammation, while monocyte-derived macrophages primarily promote allergic airway inflammation (Zasłona et al., 2014; Lee et al., 2015; Draijer and Peters-Golden, 2017). Whether the Ma2 macrophage subpopulation possesses a similar tissue-resident phenotype and exerts similar inhibitory effects on allergic airway inflammation remains to be elucidated.

Finally, we found that iron levels were reduced in the airways of the allergic asthmatics, which may be due to the decreased expression of SLC40A1 rather than elevated expression of iron accumulation molecules TFRC1 and DMT1. This differs from previous research on adults, showing that reduced iron levels in asthmatic patients are mainly attributable to TFRC1 overexpression (Ali et al., 2020). These inconsistent results may be due to several factors. Firstly, the altered iron levels in our study were observed in children with allergic asthma, while the previous study focused on all asthmatic patients, which may be influenced by population heterogeneity. Secondly, the T2-high phenotype accounts for more than 80% of all childhood asthma cases, but only about 50% of adult asthma cases (Komlósi et al., 2022). Even under the T2-high asthma phenotype, underlying endotypes differ between child and adult asthmatic patients (Kuruvilla et al., 2019), suggesting potential mechanistic differences between adult and childhood asthma. Thus, additional research is needed to clarify these discrepancies.

This study has several limitations. Although we integrated single-cell and bulk transcriptomic datasets to comprehensively profile IMR genes involved in childhood allergic asthma, our single-cell transcriptomic analysis was built on healthy samples, and we could not fully explore the disease state mechanisms. To address this, we applied deconvolution analysis with bulk transcriptomic datasets to infer changes in the cellular composition of airways in children with allergic asthma. However, our results were not completely coincident across datasets, which could be attributed to two reasons. First, inferring cell composition with bulk transcriptomic data may be less precise than that with scRNA-seq. Second, the heterogeneity in methodology, population, and underlying disease states among patient cohorts may have contributed to the observed discrepancies. Thus, further single-cell RNA-seq studies on asthmatic children with more precise exploration of cellular heterogeneity in asthmatic airways are warranted. In addition, while the potential roles of SLC40A1 and ETV5 were explored, further in-depth study is required, and the corresponding results need to be verified by further biological experiments. Finally, although our study implicated the dysregulation of iron metabolism in childhood allergic asthma, the underlying signaling pathways and the specific roles of critical cell populations (e.g., Ma2 macrophage subset) in the regulation of iron homeostasis need to be elucidated.

In summary, we identified aberrant gene expression profiles of IMR genes in childhood allergic asthma through bulk transcriptomic analysis. Of note, SLC40A1 was highly correlated with T2 airway inflammatory markers. Single-cell transcriptomic analysis identified a distinct macrophage subpopulation driving SLC40A1 expression and indicated a possible role of SLC40A1 at single-cell resolution. Our results also showed that SLC40A1 may be involved in changes in airway iron levels in children with allergic asthma. Additional in-depth research on childhood asthma should be conducted to validate our findings.

## Data Availability

The original contributions presented in the study are included in the article/Supplementary Material, further inquiries can be directed to the corresponding author.
