# Supplementary material for: Transcriptomic analysis identified SLC40A1 as a key iron metabolism-related gene in airway macrophages in childhood allergic asthma
Source: Front Cell Dev Biol. 2023 Apr 13;11:1164544. doi: 10.3389/fcell.2023.1164544 (PMC10133523; doi:10.3389/fcell.2023.1164544)

Supplementary File 1

**Table S1. Demographic information for study participants**

| **Information** | **Controls** | **Allergic**  **asthma** | **Non-allergic**  **asthma** |
| --- | --- | --- | --- |
| No. of participants | 18 | 15 | 13 |
| Age (y), mean (SD) | 3.7 (3.6) | 5.4 (3.0) | 6.2 (3.6) |
| Sex, male (%) | 61.1 | 66.7 | 53.8 |
| Past wheeze (%) | 11.1 | 100 | 100 |
| % Eosinophil, mean (SD) | 1.3 (0.8) | 2.9 (2.7) | 2.0 (1.7) |
| History of anaphylaxis (%) | 0 | 26.7 | 15.4 |
| Positive specific IgE (%) | NA | 66.7 | 23.1 |
| Eczema (%) | 27.8 | 73.3 | 40.0 |
| Parental asthma (%) | 0 | 20.0 | 0.08 |

IgE, immunoglobulin E; NA, not applicable; SD, standard deviation; y, year.

**Table S4. Data cohort characteristics.**

| **Dataset** | **CA (N)** | **HC (N)** | **Data type** | **Samples** | **Platform** | **Year** |
| --- | --- | --- | --- | --- | --- | --- |
| GSE152004 | 441 | 254 | mRNA | Nasal cells | Illumina HiSeq 2000 | 2020 |
| GSE65024 | 36 | 33 | mRNA | Nasal cells | Agilent-028004 SurePrint G3 Human GE 8x60K Microarray | 2015 |
| GSE19187 | 13 | 11 | mRNA | Nasal cells | Affymetrix Human Gene 1.0 ST Array | 2012 |
| FigShare.  14938755 | NA | 18 | mRNA | Nasal cells | Illumina NovaSeq 6000 | 2021 |

CA, childhood asthma; HC, healthy control; NA, not applicable.

**Table S5A. Primer sequence for qRT-PCR analysis**

| **Gene** | **Primers** | **Sequence (5’→3’)** |
| --- | --- | --- |
| SLC40A1 | Forward primer | TGGATGGGTTCTCACTTCCTG |
|  | Reverse primer | GTCAATCCTTCGTATTGTGGCAT |
| TFR1 | Forward primer | GGCTACTTGGGCTATTGTAAAGG |
|  | Reverse primer | CAGTTTCTCCGACAACTTTCTCT |
| DMT1 | Forward primer | ATCGGCTCAGACATGCAAGAA |
|  | Reverse primer | TTCCGCAAGCCATATTTGTCC |
| GAPDH | Forward primer | GTCTCCTCTGACTTCAACAGCG |
|  | Reverse primer | ACCACCCTGTTGCTGTAGCCAA |

qRT-PCR, quantitative reverse transcription-polymerase chain reaction.

**Table S5B. Procedure for qRT-PCR**

| **Temperature** | **Time** | **Cycle (s)** |
| --- | --- | --- |
| 95℃ | 2 minutes | 1 |
| 95℃ | 10 seconds | 39 |
| 59℃ or 65℃ | 30 seconds |
| Melt Curve 65℃ to 95℃ increment 0.5℃ | 5 seconds | 1 |
| END | | |

qRT-PCR, quantitative reverse transcription-polymerase chain reaction.

**Figure S1. Identification of T2-high and T2-low asthmatic children in GSE152004 via weighted gene co-expression network analysis.** (A) Analysis of the scale-free fit index and the mean connectivity for various soft threshold powers (β). The cut-off for soft-threshold β was set to be 0.9 and β = 9 was selected. (B) The co-expression network we constructed met the requirements of scale-free topology. (C) The clustering dendrogram. Each color represents a specific co-expression module. In GSE152004, a total of 56 gene modules were detected. (D) Hierarchical clustering of all asthma patients using 30 genes in the maroon module revealed grouping of T2-high asthma patients from T2-low asthma patients. Hierarchical clustering was performed using the “ward.D” method and “Euclidean” distance. The left cluster demonstrated grouping of T2-low asthma patients together characterised by reduced expression of CLCA1, CST1, and POSTN. The right cluster, consisting of T2-high asthma patients, was characterised by increased POSTN, CLCA1, and CST1 expression.


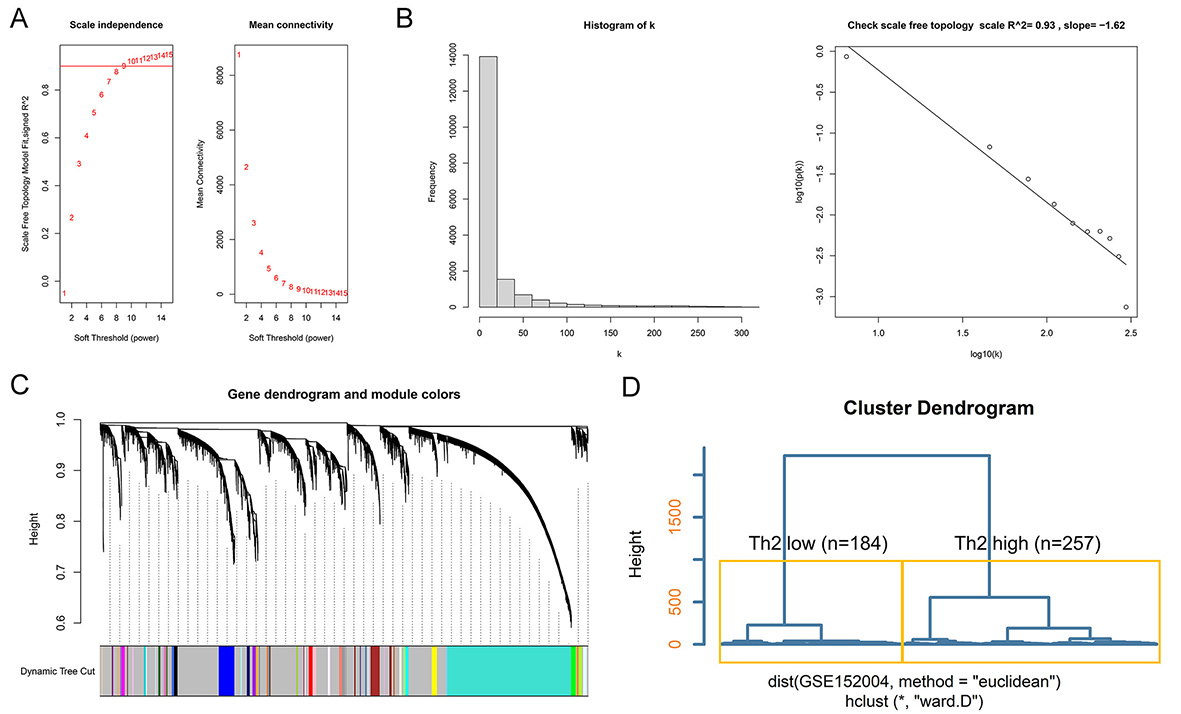


**Figure S2. Quality assessment of scRNA-seq data and dotplots of cell type marker genes.** (**A**) The genes (features), counts, and mitochondrial gene percentage of each sample. Dot plots depicting average and percent expression of genes used to classify **(**B**)** immune and **(C)** epithelial cells. The colour key from gray to purple indicated low to high expression levels. The dot size indicated the percentage of cells that expressed genes. scRNA-seq, single-cell RNA sequencing.


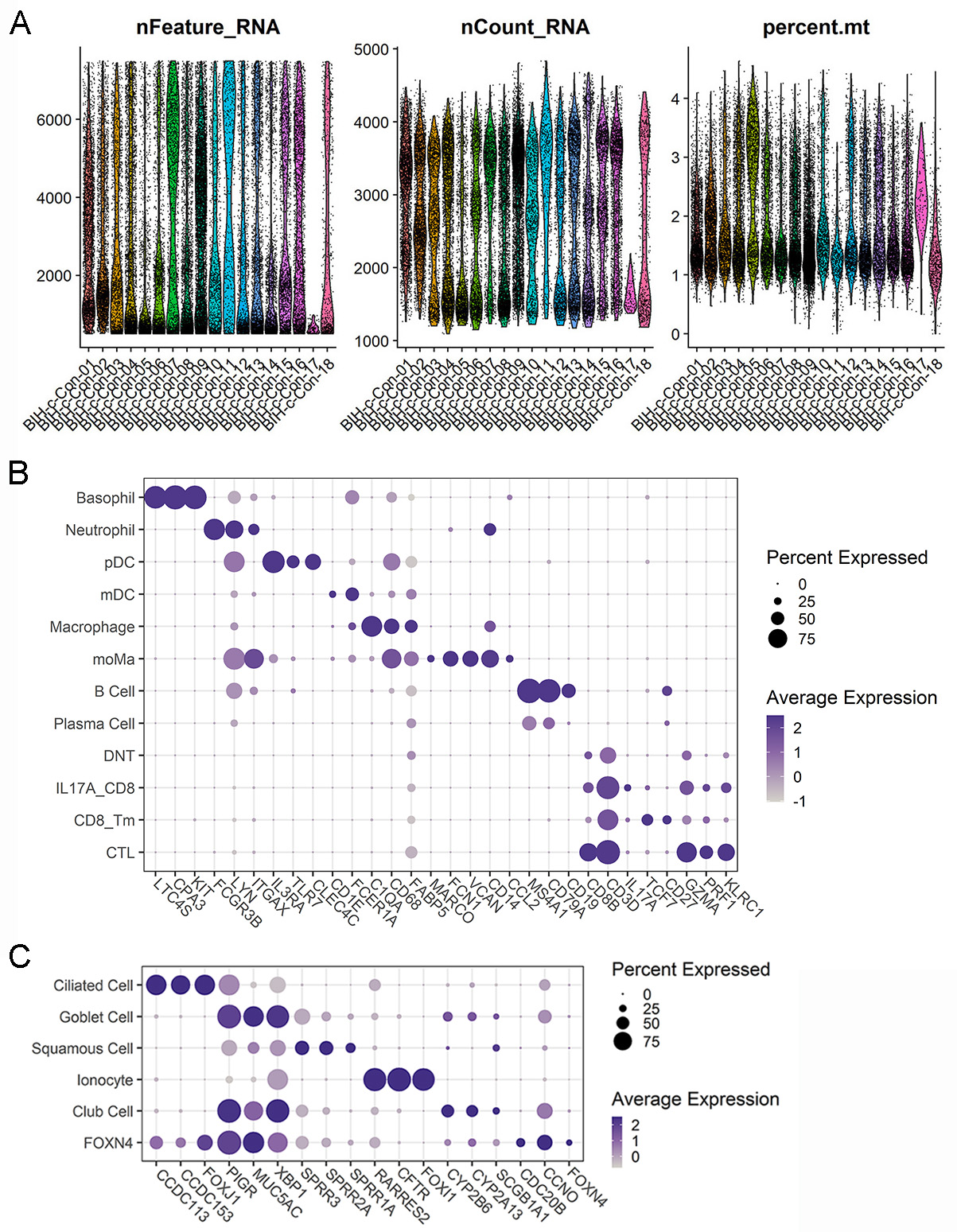


**Figure S3. Using common iron metabolism-related DEGs could distinguish from children allergic asthma with healthy subjects.** (A-C) Principal component analyses of controls and allergic asthma patients based on the five common iron metabolism-related DEGs across all datasets. X and Y axes show principal component 1 (PCA1) and principal component 2 (PCA2) that explain the percentage of the total variance, respectively. (D-F) ROC curves derived from logistic regression analyses incorporating all five common iron metabolism-related (IMR) DEGs across all datasets. AUC, area under the ROC curve; DEGs, differentially expressed genes; ROC, receiver operating characteristic.


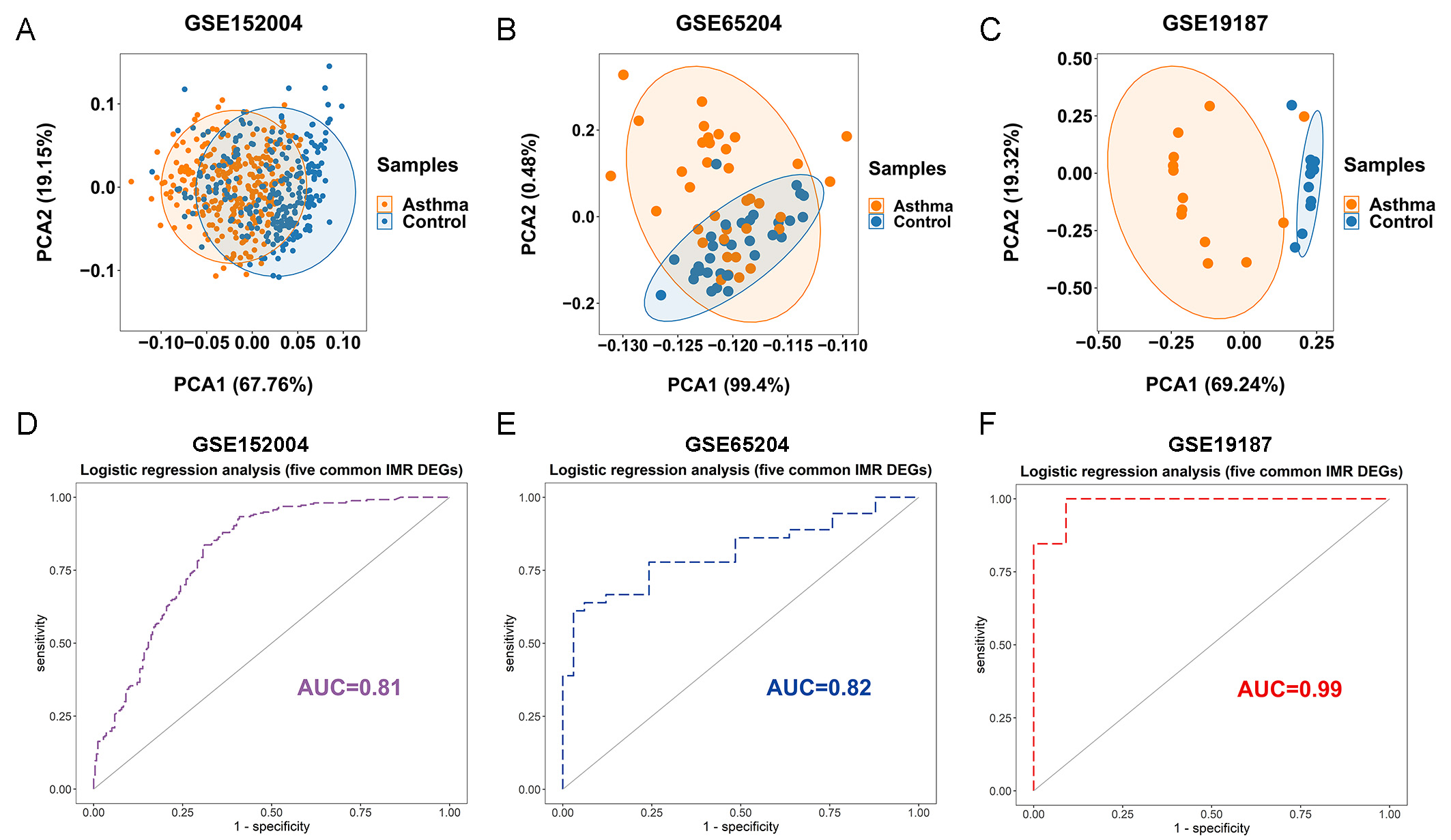


**Figure S4. Immunofluorescence staining of BAL cells from childhood** **allergic asthmatics and controls.** Representative immunofluorescent images of BAL cells stained with anti-CD68 antibody and **(A)** anti-TFR1, and (B)
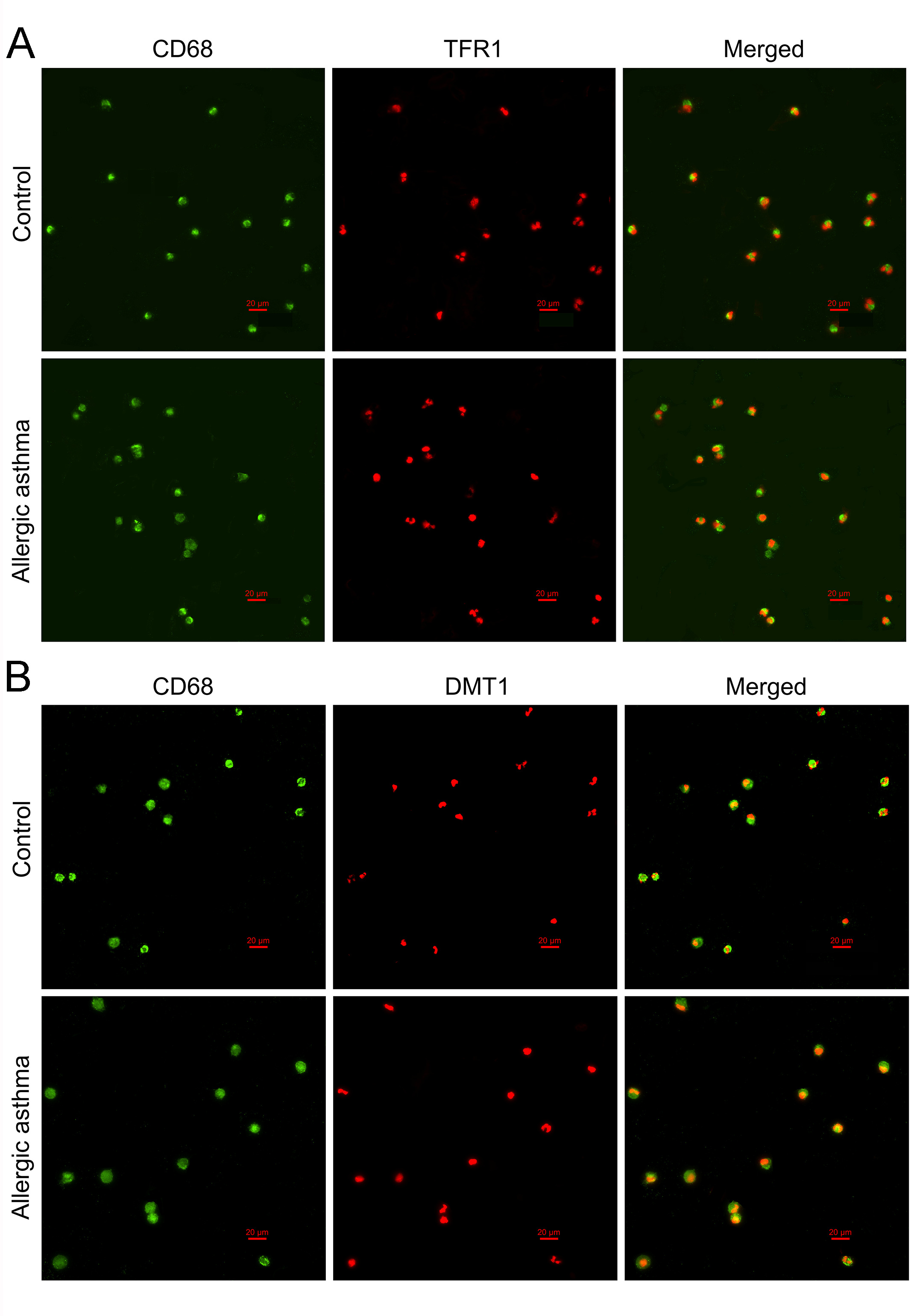
anti-DMT1antibodies. BAL, bronchoalveolar lavage.

**Figure S5. The mRNA levels of iron metabolism-related genes in childhood asthma patients and controls. (A)** TFR1, and **(B)** DMT1 showed no significant differences in expression levels between the groups. Statistical significance was assessed using Kruskal-Wallis test with Dunn’s multiple comparisons.


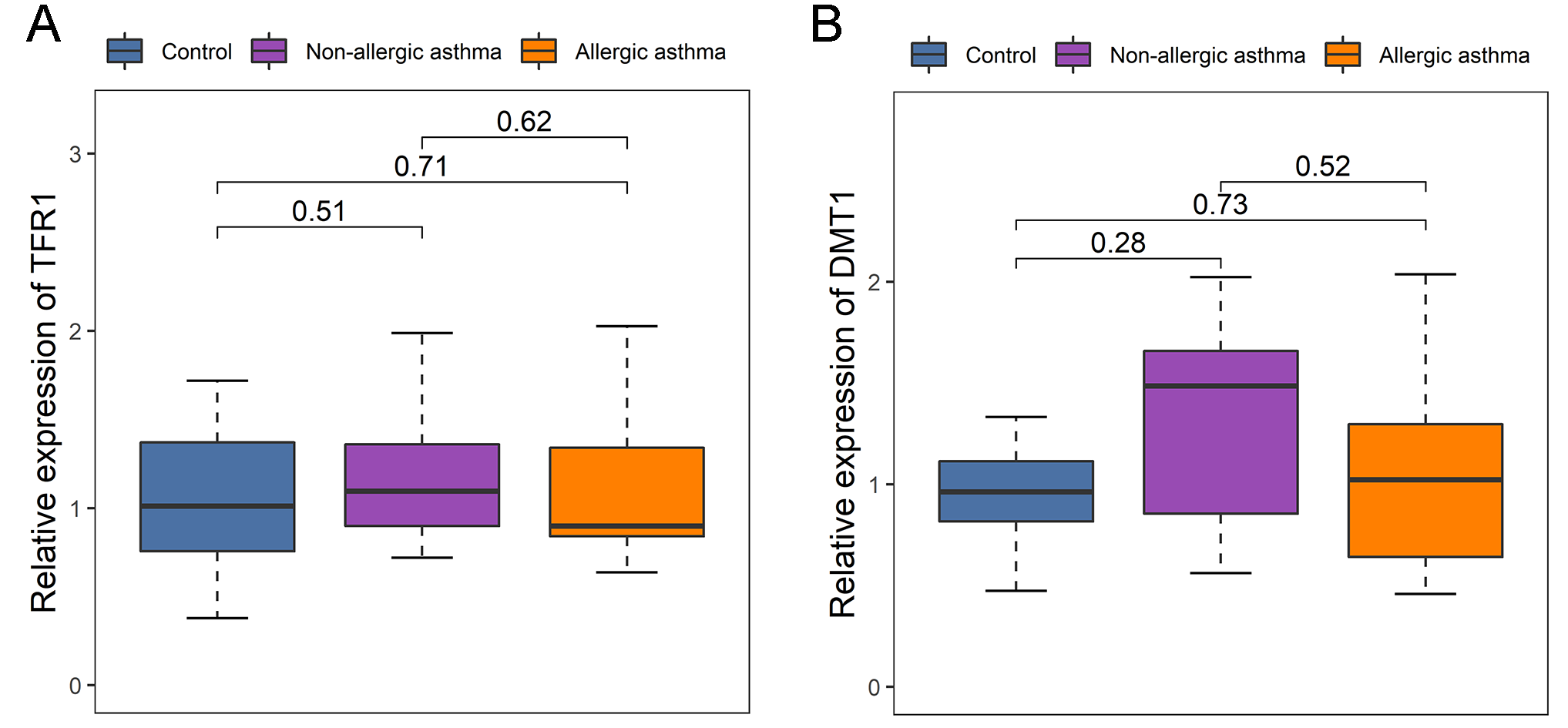

Supplement: Supplementary file 3 [file Table1.DOC]
